# An automatic drug injection device with spatial micro-force perception guided by an microscopic image for robot-assisted ophthalmic surgery

**DOI:** 10.3389/frobt.2022.913930

**Published:** 2022-08-03

**Authors:** Zhen Li, Pan Fu, Bing-Ting Wei, Jie Wang, An-Long Li, Ming-Jun Li, Gui-Bin Bian

**Affiliations:** ^1^ School of Electronic and Information Engineering, Tongji University, Shanghai, China; ^2^ State Key Laboratory of Management and Control for Complex Systems, Institute of Automation, Chinese Academy of Sciences, Beijing, China; ^3^ School of Automation, Beijing Information Science and Technology University, Beijing, China

**Keywords:** microscopic image guidance, automatic injection device, spatial micro-force perception, robot-assisted surgery, retinal vessel cannulation, *in vitro* trial

## Abstract

Retinal vein injection guided by microscopic image is an innovative procedure for treating retinal vein occlusion. However, the retina organization is complex, fine, and weak, and the operation scale and force are small. Surgeons’ limited operation and force-sensing accuracy make it difficult to perform precise and stable drug injection operations on the retina in a magnified field of image vision. In this paper, a 3-DOF automatic drug injection mechanism was designed for microscopic image guiding robot-assisted needle delivery and automatic drug injection. Additionally, the robot-assisted real-time three-dimensional micro-force-sensing method for retinal vein injection was proposed. Based on the layout of three FBG sensors on the hollow outer wall of the nested needle tube in a circular array of nickel-titanium alloys, the real-time sensing of the contact force between the intraoperative instrument and the blood vessel was realized. The experimental data of 15 groups of porcine eyeball retinal veins with diameters of 100–200 μm showed that the piercing force of surgical instruments and blood vessels is 5.95∼12.97 mN, with an average value of 9.98 mN. Furthermore, 20 groups of experimental measurements on chicken embryo blood vessels with diameters of 150–500 μm showed that the piercing force was 4.02∼23.4 mN, with an average value of 12.05 mN.

## 1 Introduction

Retinal vein obstruction (RVO) is an important cause of vision loss in the elderly worldwide, affecting approximately 16.4 million people (Rogers et al., 2010). It manifests as thrombus formation in the center or branch blood vessels of the retinal vein, resulting in severe loss of vision and even blindness (Ip and Hendrick, 2018). Due to the complexity, high precision, and importance of the anatomical structure and function of the retina, retinal surgery is considered a very challenging and difficult surgical operation. Today, there is no clinically effective treatment for this disease. In recent years, retinal vein cannulation (RVC) has been proposed as an innovative procedure (Peng et al., 2017). Surgeons inject thrombolytic drugs into the blocked retinal vein to dissolve the thrombus, which is expected to restore blood circulation in the retinal vein (Kadonosono et al., 2018; Kadonosono, 2020).

**FIGURE 1:**
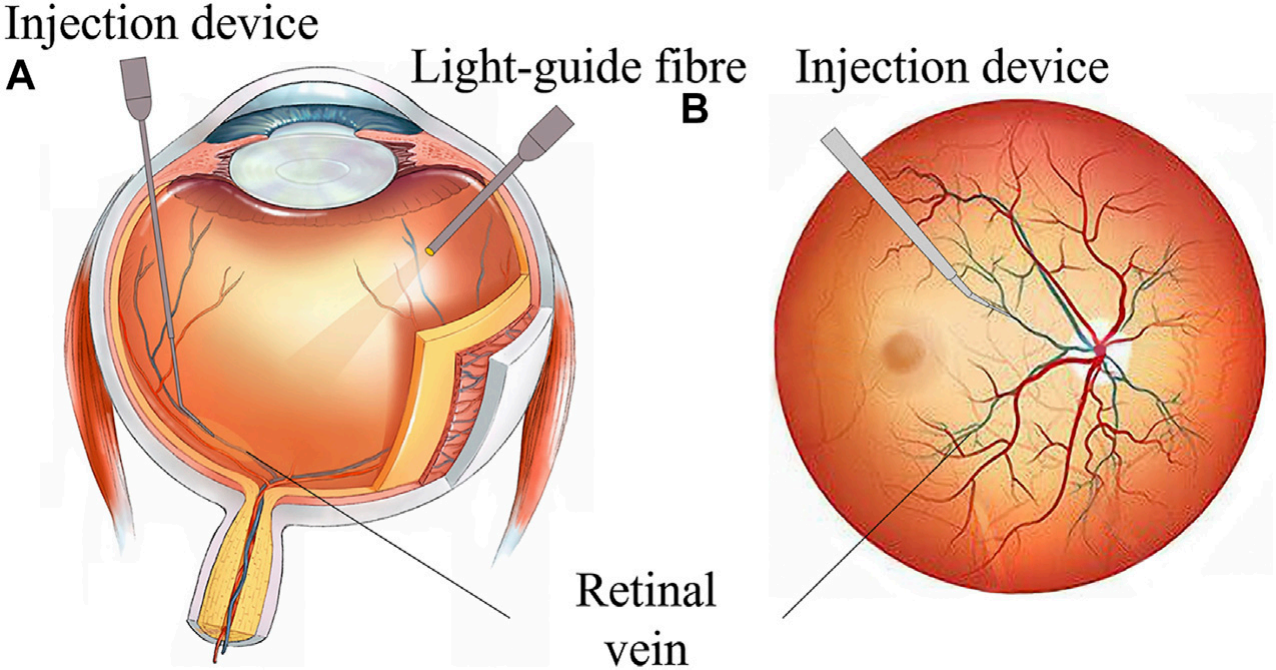
RVC surgical manipulation. **(A)** Coronal plane of the eyeball. **(B)** Transverse plane of eyeball scheme.

 However, retinal intravenous injection puts forward higher requirements for surgeons’ operations (Ahmad et al., 2020; Mishra and Ashraf, 2020). As shown in Figure 1, during the operation, the surgeon needs to hold a micro-syringe through the anterior segment and vitreous body under the visual field of the posterior segment of the microscope and then position it above the blocked retinal vein. After puncturing the blood vessel, the surgeon needs to inject the thrombolytic drug stably and slowly. The diameter of retinal blood vessels is about tens of microns to two hundred microns, and the average amplitude of the human hand tremor reaches 156 μm (Channa et al., 2017; Kasina et al., 2017), which is difficult for surgeons to meet the accuracy requirements of the surgery. In retinal surgery, 75% of the contact force between the instruments and the retinal tissue is less than 7.5 mN, and surgeons can only perceive 19% of the contact force (Kadonosono et al., 2021), which further restricts the precise implementation of the surgery. In summary, retinal vein injection and thrombolytic surgery cause the following difficulties: (1) how to deliver the device stably and accurately to the blood vessel of approximately 100 microns; (2) precisely sense the tiny contact force between the device and the tissue, reducing the chance that the surgeon may completely puncture or penetrate the blood vessel; and (3) the injection device accurately pierces through the vein wall and stays at the specified depth, maintaining a fixed posture for stable injection.

Surgical robots provide a viable solution for the precise implementation of RVC (Gerber et al., 2020b; Jara et al., 2020). Through the intelligent injection device at the far end of the robot, the surgical robot assists the surgeon in accurately delivering the intelligent injection device to the target blood vessel according to the predetermined path, measures the contact force between the device and the blood vessel tissue in real time, and steadily pushes the thrombolytic drug at the specified speed to improve the accuracy and safety of operation. Internationally, Carnegie Mellon University developed a handheld actively stable 3-DOF eye surgery micromanipulator micron, which could eliminate unconscious hand movements for precise needle insertion (MacLachlan et al., 2011). Researchers from Vanderbilt University developed a miniature distance sensor based on optical coherence tomography (OCT) technology. The sensor can be embedded in surgical forceps. During the operation, surgeons can observe the OCT image in front of the forceps in real time, thereby judging the distance of the forceps end relative to the tissue (Yu et al., 2015). Researchers at Johns Hopkins University deployed fiber Bragg grating (FBG) sensors in the surgical instrument shaft and developed, for example, surgical forceps, surgical hooks, and injection needles, which could sense the lateral force of 2-DOF (Gonenc and Iordachita, 2016; Gonenc et al., 2018; Patel et al., 2020; He C. et al., 2019; Vander Poorten et al., 2020; He et al., 2020a,b, He X. et al., 2019). Experiments were performed on self-made eyeball simulations. These instruments could be directly held by the surgeon to operate or installed at the end of the robot for master–slave operation.

Researchers at Leuven University proposed a surgical instrument design method that combines OCT technology and FBG sensors to detect distance and force information at the same time, realizing multi-information fusion detection (de Smet et al., 2016a; Gijbels et al., 2016; Willekens et al., 2017; Smits et al., 2018; Ourak et al., 2019; Schoevaerdts et al., 2019). They also proposed a new type of bio-impedance sensor to obtain the relative location of the injection needle and tissue (Schoevaerdts et al., 2018). The PRECEYES surgical robot developed by the University of Eindhoven cooperated with surgeons to inject drugs under the retina and successfully punctured live pig eyes (Meenink et al., 2012). In clinical trials, Preceyes robot-assisted surgeons successfully performed retinal surface detachment operations on six patients with fundus diseases, with an average thickness of the proliferative membrane of 61 microns, and performed subretinal injections on three patients (de Smet et al., 2016b; Edwards T. et al., 2018). In 2017, Leuven University Hospital successfully carried out the world’s first robot-assisted RVC (Edwards T. L. et al., 2018; Gijbels et al., 2018). Four patients received this technology in a phase I clinical trial, which proved the feasibility and safety of robot-assisted 100-micron intravenous cannula and 10 min anticoagulant injection to dissolve blood clots. Besides, researchers from UCLA (Chen et al., 2018; Gerber et al., 2020a), IACAS (Zhang et al., 2021b,a), and BHU (Chang-Yan et al., 2018; Han and Yang, 2019; Han et al., 2021; Han and Yang, 2021), among others (Liu et al., 2018; Sumimoto et al., 2019; Huda et al., 2020; Liu et al., 2020; Suzuki and Wood, 2020) conducted a series of trials on the precise perception and accurate manipulation of microscopic surgery.

In summary, the existing intelligent surgical instruments can realize the functions of vascular puncture and micro-force perception. However, there are still the following problems. First, as the most direct judgment condition of puncture, the axial force of the injection device is still lacking in sensing methods. Secondly, it is still necessary to manually push thrombolytic drugs. Due to the small size of the retinal vein vascular cavity and the small injection amount, the injection speed is slow and the duration is long. Limited to the physiological limits of manual operation, long-term stable delivery is difficult to achieve. The injection speed cannot be precisely adjusted. Besides, repeated disassembly of the syringe results in the incomplete sealing of the mechanism, complicated disinfection procedures, and poor bacterial isolation.

The contributions of this article are as follows. A 3-DOF automatic injection device coordinated with a remote mechanical constraint fixed point was designed to achieve robot-assisted needle delivery and automatic drug injection with adjustable speed. A three-dimensional micro-force-sensing method with the layout of the FBG sensor on the hollow outer wall of the nickel-titanium alloy on the nested needle tube is proposed to realize the real-time perception of the contact force between the instrument and the blood vessel during the operation. Robot-assisted repeated injection experiments were carried out on the isolated porcine eyeball retinal vein and chicken embryo blood vessel. The results showed that the device designed in this study could detect the time of puncturing the blood vessel more sensitively and stably inject the tissue-type plasminogen activator (t-PA) solution into the blood vessel. Through the design of the automatic push mechanism, the axial force-sensing method, and the configuration of the ophthalmological surgery robot system, the problems of drug injection speed control and puncture sensitivity are solved, and the automation degree of ophthalmological surgery is improved. It improves the safety and accuracy of the operation and provides a reference for the clinical trial of robot-assisted microvascular injection.

The remainder of this study is organized as follows: Section 2 describes the mechanical design principle and finite element calculation of the 3-DOF three-dimensional force-sensing automatic injection device. In Section 3, the force calibration method is provided, and the three-direction force calibration results are conducted. Section 4 explained the conducted experiments and the results of the porcine eyeball and chicken embryo puncture and injection. Finally, in Section 5, a conclusion is made to summarize the feasibility and effectiveness of the proposed methods, including the next research direction.

## 2 3-DOF three-dimensional force-sensing automatic drug injection device

The dual-arm robot equipped with the mechanism of mechanically constraining the distal center fixed point can accurately position the end of the medicine injection device to the corneal incision. For the most critical vascular penetration moment, real-time contact force perception is an important basis for judgment. According to the operational requirements of the procedure, the injection device needs to realize the functions of needle rotation alignment, linear feed puncture, and automatic drug advancement. The pushing DOF by the instrument can deliver the sleeve to the vicinity of the target retinal vein. Then, the high-precision retinal vein puncture and the stable injection of thrombolytic drugs are realized by the multi-stage nested injection needle tip.

### 2.1 Design of miniaturized 3-DOF drive mechanism

**FIGURE 2:**
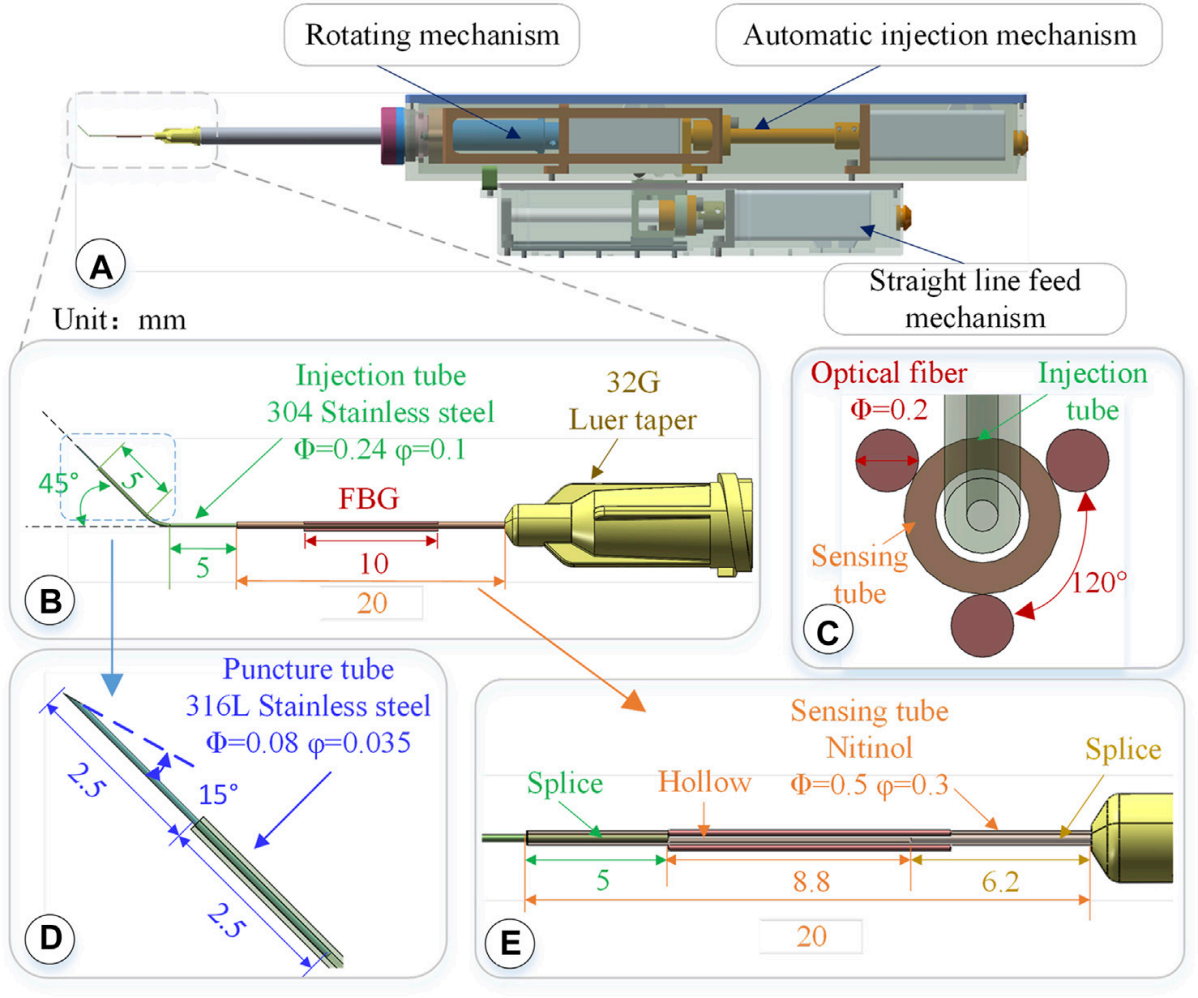
3-DOF micro-force-sensing medicine injection device. **(A)** 3-DOF micro-force-sensing medicine injection device driving mechanism. **(B)** 3-DOF micro-force-sensing sleeve needle. **(C)** FBG sensor cross section array layout. **(D)** Puncture needle. **(E)** FBG sensor axial direction layout position.

The driving mechanism is composed of three parts: a linear feeding mechanism, a rotating mechanism, and an automatic injection mechanism. The designed length is 260 mm and the height is 65 mm, as shown in Figure 2A. The internal packaging is complete, and the external parts have a quick-fix and locking interface, which can be disassembled and used repeatedly. The rotation alignment DOF of the drive mechanism is realized by a stepping motor, the needle rotation range is 360°, and the accuracy is 0.05°. It is used to align the sleeve needle with the puncture point along the axial direction of the blood vessel.

The linear puncture feeding mechanism uses a stepping motor to drive the precision lead screw to rotate and then drive the bearing on the lead screw to perform a linear motion, achieving high-precision linear puncture movement along the axial direction of the sleeve. Its pushing stroke is 45 mm, with an accuracy of 5 μm. After the blood vessel is punctured and the drug injection is completed, the sleeve needle can be withdrawn. The automatic injection module is composed of a stepping motor and a high-precision screw, which is driven by the back-end motor and then transferred to the front end to push the plunger of the syringe to realize the injection function. These three mechanisms use the smallest possible stepper motor (Eastern Motor, ARM14SA0K 20*20*64 mm) under the condition of ensuring the driving force and stroke. The microsyringe is fixed at the end of the rotating alignment mechanism by a seal capping. Micro-syringes of different specifications can be replaced according to the amount of medicine injected. In the light of the clinical dosage of 0.3–0.6 mg/kg t-PA drug (Novoprotein Scientific Inc.), a 50–100 μL syringe is used in this article.

### 2.2 Structure design of three-dimensional micro-force-sensing injection sleeve

The diameter of retinal veins is usually less than 150 μm. During injection, the needle tip is delivered close to the axis of the blood vessel. Therefore, higher requirements are put forward on the design of the sleeve structure. In this study, the finite element calculation is used to obtain the deformation distribution of the sleeve structure after the needle tip is stressed. The sleeve is designed as a circular array layout of FBG sensors, placing the FBG sensor near the tip of the needle where the deformation is greatest to improve the accuracy and sensitivity of force perception. The sleeve can be used as a disposable consumable. Common clinical low-temperature disinfection and sterilization methods (such as formaldehyde steam fumigation) can be used for surgical treatment.

#### 2.2.1 Structure design of three-level nested mixed material injection sleeve

The injection sleeve adopts a cascade structure design and consists of three sleeve needle tubes of different materials and sizes. The three needle tubes share the same center, using a medical device biocompatible optically transparent adhesive (UV glue) to bond two by two, as shown in Figure 2B.The function of the puncture needle tube is to pierce through the blood vessel wall and enter the blood vessel. It remains stable in the blood vessel for a period of time till the drug injection is completed. The puncture needle tube is made of 316L stainless steel, which has higher strength, corrosion resistance, and biocompatibility. As shown in Figure 2D, the outer diameter of the puncture needle tube is 80 μm, and the inner diameter is 35 μm. It could be used to inject retinal vessels with a minimum diameter of 100 μm. The needle part adopts laser cutting to make a 15°bevel, which could reduce the contact area and force area to increase the pressure. At the same time, the penetration stroke is increased to improve safety, making it easier for the needle tip to pierce through the blood vessel wall. The length of the puncture needle is 5 mm, and the inclined surface also effectively limits the pressure of the liquid in the narrow lumen, which is conducive to drug release. The puncture needle tube is inserted into the injection sleeve at 2.5 mm and glued. The outer diameter of the injection sleeve is 0.24 mm, the inner diameter is 0.1 mm, and the length is 13 mm. The material is 304 stainless steel. The injection cannula is pre-bent with 45°, which facilitates the formation of a reasonable angle between the puncture needle tube and the blood vessel, reduces the phenomenon of double puncture, and increases the visibility of the injection sleeve structure under the microscope. The injection sleeve is inserted into the head of the sensing sleeve at 5 mm and bonded, as shown in Figure 2E. The outer diameter of the sensing sleeve is 0.5 mm, the inner diameter is 0.3 mm, and the length is 20 mm. The needle tube is made of nickel-titanium alloy with a smaller elastic model (67 MPa of Nitinol, 216 MPa of 304 stainless steel). The end of the sensing sleeve is inserted into a 32G (outer diameter of 240 μm, inner diameter of 100 μm) medical syringe head for bonding so that both ends of the sensing sleeve are fixed and hollow.

#### 2.2.2 Three-dimensional micro-force sensor design and layout

The real-time interactive force perception under robot-assisted surgery can effectively avoid the rupture of the blood vessel caused by the secondary puncture and improve the safety of the operation. The human retina is composed of 10 layers from the outside to the inside, and its thickness is less than 0.5 mm. It is the part of the eye that converts light signals into nerve signals. When the retina is subjected to excessive force, its fine structure may be destroyed, causing irreversible tissue damage. Moreover, the interaction force of these fine organizations is often lower than the threshold of human perception. The diameter of retinal blood vessels is smaller than the physiological limit of human handshaking. Therefore, three FBGs are arranged on the nickel-titanium alloy sleeve in a uniform annular array. Through the tip interactive force transmission, the nickel-titanium alloy sleeve is deformed, producing the change of grating period. Then, the deformation is calculated according to the change in the center wavelength of the reflected light, and the end force can be calculated. The small size, good biocompatibility, and immunity to electromagnetic interference of FBG sensor make it easier to use *in vivo*.

Three FBG sensors are pasted on the hollow outer wall of the sensing sleeve with a medical epoxy adhesive, as shown in Figure 2C. The length of the grid area of the FBG sensor is 10 mm, and the outer diameter is 0.2 mm, evenly distributed at 120° along the central axis of the sleeve. Deformation occurs together with the sensing needle tube, which measures the contact puncture force between the needle tip and the blood vessel. According to the principle of the FBG sensor, the wavelength of the reflected light of the FBG sensor is affected by the strain and temperature of the grid area independently. The FBG sensor reflected light wavelength change Δ*λi* can be expressed as follows:

$$\Delta \lambda_{i}=\lambda_{i}-\lambda_{0}=k_{\varepsilon }\varepsilon +k_{T}\Delta T.$$ (1)

In the formula, k*ɛ* and *k*_*T*_ are the influence coefficients of deformation and temperature changes on the wavelength caused by the reflected light by the FBG sensor, determined by the inherent properties of the material.

When the end of the injection device is close to the blood vessel area, the FBG sensor is inside the eyeball. As the temperature inside the eyeball is usually maintained at a constant level, the wavelength change of the reflected light is only related to the strain of the grating. The FBG sensor is pasted on a sleeve made of nickel-titanium alloy, of which one end is fixed and the other is stressed. After the force end is deformed by force transmission, strain occurs together with the FBG gate area. According to Hooke’s law, the axial component force of the needle tip has a linear relationship between strain and stress. The mechanical model of the radial force component is equivalent to the cantilever beam model, and the strain *ɛ* is generated on the cross section of the grating region:

$$\varepsilon =\frac{\sigma }{E}=\frac{M\text{y}}{IE}=\frac{FL\text{y}}{IE}={\text{k}}_{\text{0}}F,\text{therein},{\text{k}}_{\text{0}}=\frac{L\text{y}}{IE},$$ (2)

where *σ* is the cross-sectional strain of the grating region. *E* is the elastic modulus of the Nitinol sleeve. *I* is the moment of inertia of a circular section. *M* is the torque experienced by the sleeve. *y* is the distance from the surface to the neutral plane. *L* is the distance between the tip of the needle and the cross-section of the grating grid area. *F* is the radial component force of the needle tip. *k*_0_ is a constant, which is related to the material and geometric properties of the nickel-titanium alloy sleeve. Therefore, the strain generated by the FBG sensor has a linear relationship with the radial component force of the needle tip. The force of the needle tip determines the amount of change in the wavelength of the reflected light of the FBG sensor. The FBG sensor is in a 120° circular array distributed configuration, and each sensor can perform micro-force sensing:

$$\left[\begin{array}{c} \Delta \lambda_{1}\\ \Delta \lambda_{2}\\ \Delta \lambda_{3} \end{array}\right]=\left[\begin{array}{ccc} K_{11} & K_{12} & K_{13}\\ K_{21} & K_{22} & K_{23}\\ K_{31} & K_{32} & K_{33} \end{array}\right]\left[\begin{array}{c} F_{x}\\ F_{y}\\ F_{z} \end{array}\right],$$ (3)

where Δ*λ*_1_, Δ*λ*_2_, and Δ*λ*_3_ are the wavelength changes of the reflected light from the three FBG sensors. *K*_*ij*_ is the element of corresponding coefficient matrix of three FBG sensors. *F*_*x*_, *F*_*y*_, and *F*_*z*_ are the force applied by the needle tip in the axial and radial directions. Using the generalized inverse matrix of the coefficient matrix *K* and the wavelength change of the reflected light of the FBG sensor, the force on the needle tip can be calculated as

$$\left[\begin{array}{cc} & F_{X}\\ & F_{Y}\\ & F_{Z} \end{array}\right]=K^{+}\left[\begin{array}{cc} & \Delta \lambda_{1}\\ & \Delta \lambda_{2}\\ & \Delta \lambda_{3} \end{array}\right].$$ (4)

During the operation, the contact force F between the needle tip and the tissue can be decomposed into

$$F=\sqrt{F_{\text{x}}^{2}+F_{y}^{2}+F_{\text{z}}^{2}}.$$ (5)

## 3 Simulation and calibration of three-dimensional micro-force-sensing injection sleeve

The structure of the three-level nested mixed material injection sleeve is shown in Figure 2B, which is locked on the micro-syringe by the Luer head. The length is 32 mm, which meets the required length of fundus injection. The sleeve structure is easy to replace, and the sleeve and micro-syringe can be replaced after the surgical operation, making it more in line with the convenient operation habits of doctors and the clinical needs of bacteria isolation.

**FIGURE 3:**
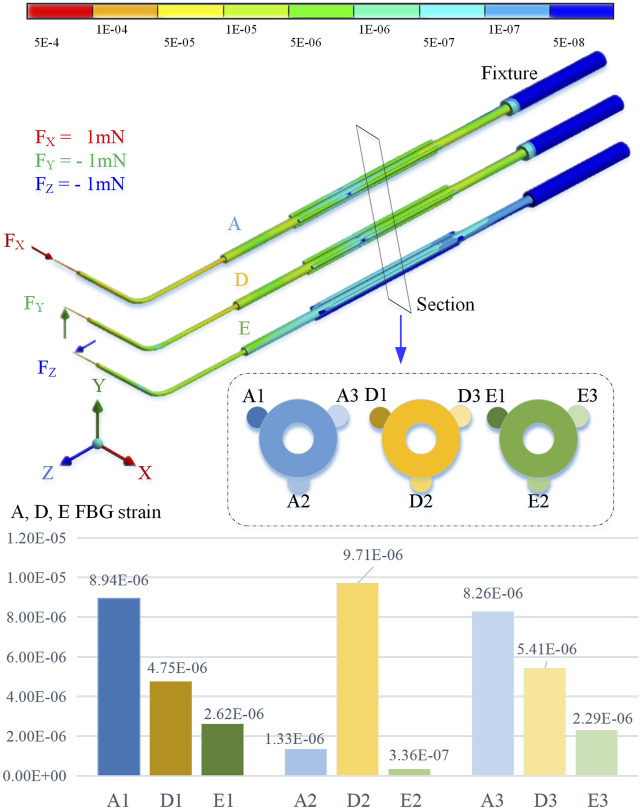
Three-direction force FEM simulation calculation results.

By using the finite element method for stiffness calculation and stress-strain numerical calculation, along the cross section of the sleeve axis from 10 o’clock, it is marked as the No. 1 position and the counterclockwise interval is 120° as the No. 2 and No. 3 positions, respectively. By applying an extrusion force of 1 mN to the end of the sleeve in the *X*, *Y*, and *Z* directions, the strain at the three sensing positions is shown in Figure 3.

The simulation calculation results show that when the axial pressure *F*_*x*_ is applied, the strains at No. 1 and No. 3 positions are significantly greater than those at No. 2. When the radial force *F*_*y*_ is applied, the deformation of No. 2 position is greater than No. 1 and No. 3. When the radial force *F*_*z*_ is applied, the deformation at the No. 1 and No. 3 positions is smaller, but it is significantly higher than the No. 2 position.

### 3.1 Calibration method

In order to determine the relationship between the wavelength change of the reflected light by the FBG sensor and the contact force of the needle tip, it is necessary to calibrate the three-dimensional micro-force-sensing injection sleeve.

**FIGURE 4:**
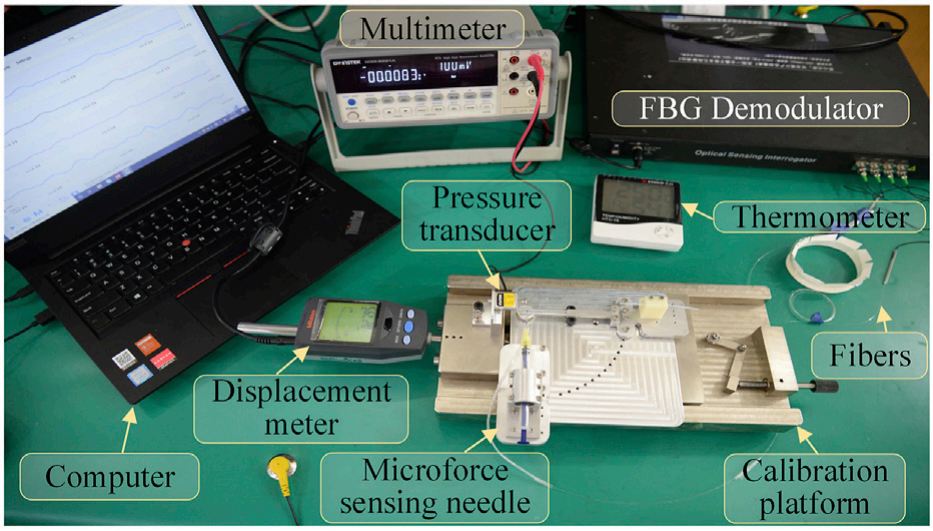
Composition of micro-force calibration platform for the injection device.

The designed 3D micro-force calibration platform is shown in Figure 4. The calibration platform consists of a fiber grating demodulator (Beijing TONGWEI SENSING, TV125-16), pressure sensor (FUTEK, S strain gauge force sensor LSB200, range 1 N, sensitivity 2 mV/V), and an independently designed and manufactured linear displacement platform (range 2 mm, resolution 1 μm), digital multimeter (GWINSTEK, GDM-9061, DCV accuracy 0.0035%), and thermometer.

The pressure sensor is mounted on the fixed end of the displacement platform by a fixture. The three-dimensional force-sensing injection sleeve is fixed to the mobile end of the displacement platform. By adjusting the posture through different fixtures, it can be subjected to forces from different directions. The digital multimeter receives the voltage signal output by the pressure sensor, calculating the force value of the pressure sensor by measuring the voltage value.

The three channels of the fiber grating demodulator are connected to the three FBG sensors arranged in an array on the three-dimensional micro-force-sensing injection sleeve to measure the change of its reflected wavelength. The fourth channel is connected to a vacant FBG sensor to measure the reflected wavelength change caused by temperature in the experimental area. Exerting a force of 0–20 mN in one direction to the injection sleeve with a step of 1 mN, the wavelength change of the reflected light from the FBG sensor is recorded. By substituting the coefficient matrix, the relationship between the force of the injection sleeve and the wavelength change of the feedback light by the three FBG sensors can be obtained.

### 3.2 Calibration experiment

**FIGURE 5:**
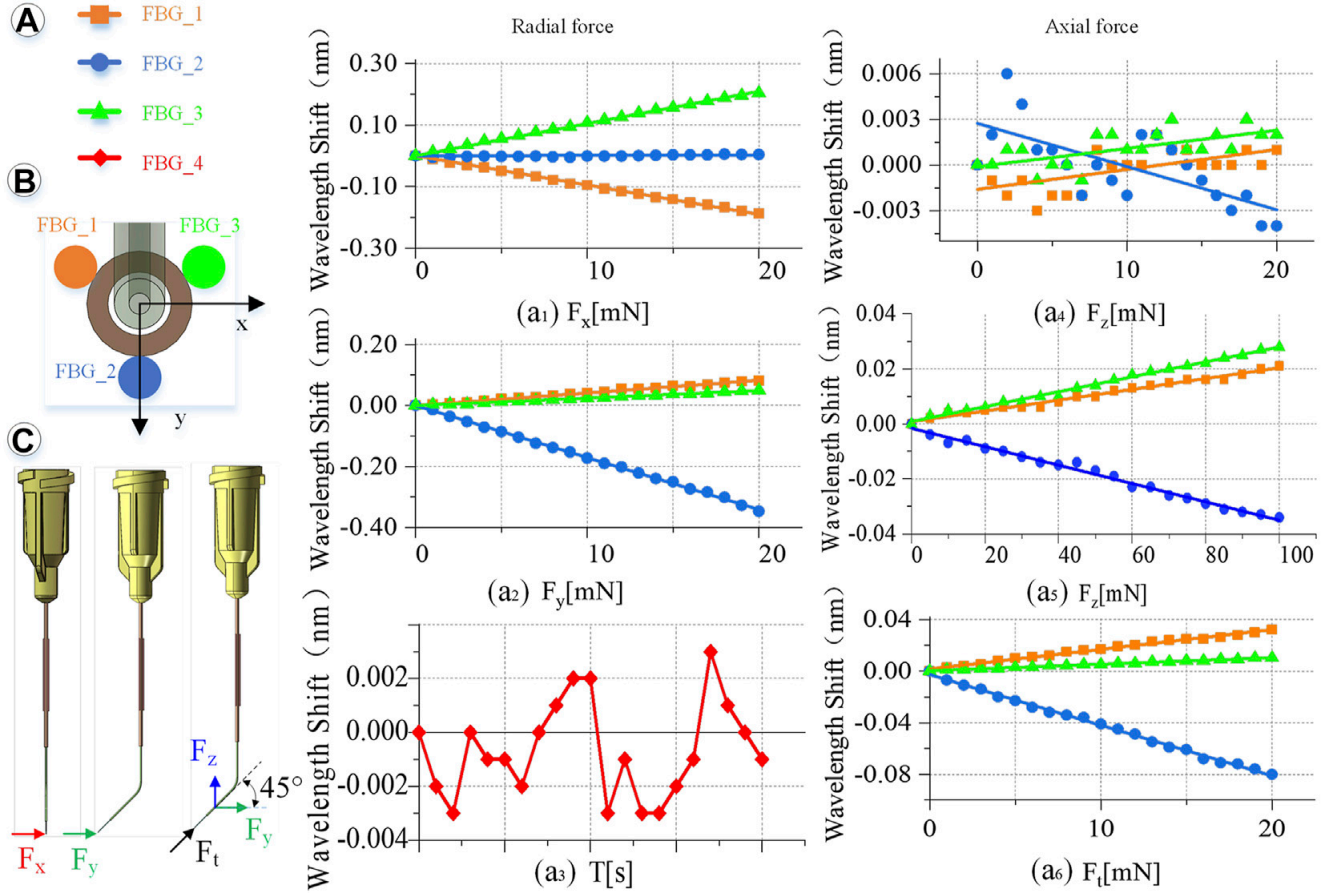
Calibration experiment results. **(A)** Wavelength change of the reflected light of the four FBG sensors. (a_1_) When *F*_*x*_ is 0–20 mN. (a_2_) When *F*_*y*_ is 0–20 mN. (a_3_) Influence of temperature on the feedback wavelength change of FBG sensor during the experiment. (a_4_) When *F*_*z*_ is 0–20 mN. (a_5_) When *F*_*z*_ is 0–100 mN. (a_6_) When *F*_*t*_ is 0–20 mN. **(B)** FBG sensor number and layout. **(C)** Direction of the force on the end of the injection sleeve.

Keep the indoor temperature constant during calibration and the fourth FBG sensor vacant without external force. Its wavelength change is only affected by temperature. By observing the wavelength change during the calibration experiment, the maximum is 0.004 nm. Therefore, the influence of temperature on the sensor can be ignored, due to a difference of more than 10 times the relative value, as shown in Figure 5A. Set the transverse direction of the radial force in the *X* direction, a clockwise rotation of 90° is the *Y* direction, and the right-hand coordinate and axial force are the *Z* direction, as shown in Figure 5B.

Radial force (*X*, *Y* direction) calibration is conducted as shown in Figure 5C. Fix the injection sleeve to the calibration platform. The needle tip is perpendicular to the pressure sensor as a whole. Therefore, it is only subjected to the *X* direction force, *F*_*x*_. Then, the demodulator is reset to zero, and the linear displacement platform is adjusted to make the injection sleeve close to the pressure sensor. Next, increase the needle load from 0 to 20 mN, and record the wavelength change of the reflected light from the FBG sensor demodulator every 1 mN. Finally, the injection sleeve rotates 90° counterclockwise and only receives the force *F*_*y*_ in the *Y* direction. The same method is used to record the relationship between the wavelength change of the reflected light by the FBG sensor and *F*_*y*_. The slope is the coefficients *k*_*n*1_, *k*_*n*2_, *k*_*n*3_ (*n* = 1, 2, 3) of coefficient matrix *K*.

Axial force (*Z* direction) calibration is conducted as follows. The sensing sleeve uses materials with a small elastic modulus and is designed into a special hollow structure. Thus, the deformation in the *Z*-axis direction is enlarged, making it possible to measure the force *F*_*z*_ in the *Z* direction. The simulation calculation results show that the axial force causes less influence on the deformation of the three-dimensional force-sensing injection sleeve than the radial force. By using the same method to calibrate the axial force, when the axial force is applied in the range of 0–20 mN, the linearity of the wavelength change of the reflected light by the FBG sensor is very poor as shown in Figure 5a4. Change the axial force to gradually load in the range of 0–100 mN, the wavelength change by the reflected light from the FBG sensor is recorded every 5 mN. The maximum change in the wavelength of the reflected emission light recorded by the FBG sensor is only 0.03 nm, as shown in Figure 5a5, which is far from meeting the measurement requirements.

Therefore, this study proposes a method to indirectly calibrate the axial force *F*_*z*_ under small deformation. Apply a force *F*_*t*_ to the end of the injection cannula. *F*_*t*_ can be decomposed into *F*_*y*_ and *F*_*z*_, and the angle between *F*_*t*_ and *F*_*y*_ and *F*_*z*_ is 45°. Increasing the load of the injection sleeve, the *F*_*t*_ rises from 0 to 20 mN, and the wavelength change of the reflected light from the FBG sensor is recorded every 1 mN. Repeat the calibration experiment and change the angle between *F*_*t*_ and *F*_*y*_, *F*_*z*_ every 5° to get different *F*_*t*_. The relationship between *F*_*t*_ in different directions and the wavelength change of the reflected light by the FBG sensor can be obtained.

Obtain the relationship between the different axial force and the wavelength change of the reflected light by the FBG sensor, and use the least square method to process the data.

Incorporating Formula 6 can yield *k*_*n*1_, *k*_*n*2_, *k*_*n*3_ (*n* = 1, 2, 3):

$$\lambda_{\text{n}}=F_{\text{x}}{\text{k}}_{\text{n}1}+F_{\text{y}}{\text{k}}_{\text{n}2}+F_{z}{\text{k}}_{\text{n}3},$$ (6)

Using the above data, the *K* matrix can be fitted as follows:

$$K=\left[\begin{array}{ccc} -\text{0}\text{.}0095293 & \text{0}\text{.}0041544 & \text{0}\text{.}0002074\\ \text{0}\text{.}000507 & -\text{0}\text{.}017076 & -\text{0}\text{.}003578\\ \text{0}\text{.}0104638 & \text{0}\text{.}0024557 & \text{0}\text{.}0002832 \end{array}\right].$$ (7)

### 3.3 Calibration results

Multiple measurements are taken to determine the calibration accuracy. Adjust the angle and posture of the injection sleeve to obtain more force as a measurement value. Record the wavelength change of the FBG sensor, and calculate the force value through the *K* matrix. Comparing the measured value with the actual value, the RMS error between the measured value of the force in the *X*, *Y*, and *Z* directions and the actual value is 0.24, 0.18, and 1.71 mN, respectively. The RMS errors of the radial two-dimensional and the spatial three-dimensional force are 0.3 and 1.73 mN, respectively.

## 4 Microscopic image guidance robot-assisted three-dimensional micro-force-sensing and drug injection animal experiment

**FIGURE 6:**
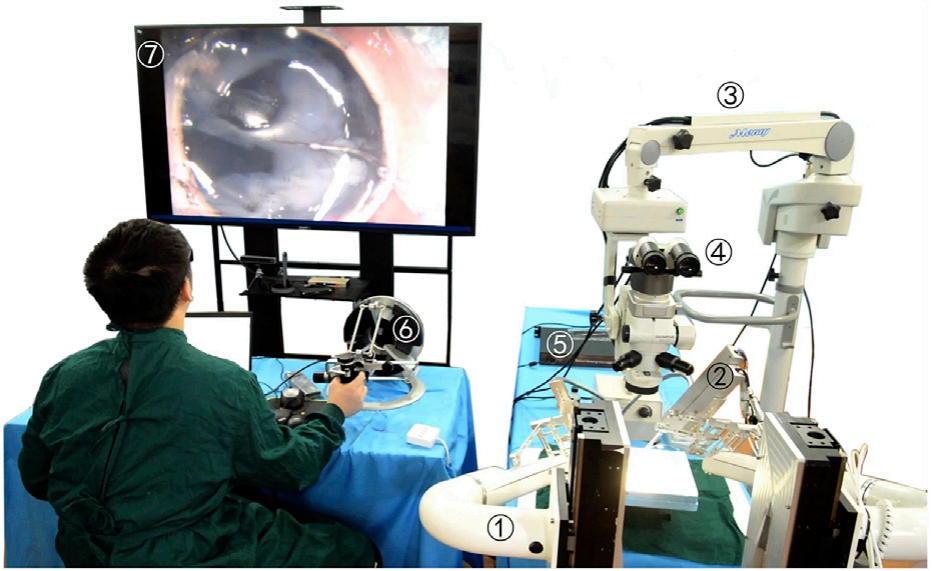
Microscopic image guidance robot-assisted pig eyeball retinal vein injection. (1) Dual-arm surgical robot. (2) Drug injection device. (3) Microscope. (4) Spectroscope and cameras. (5) FBG demodulator. (6) Omega 6. (7) 3D microscopic image.

**FIGURE 7:**
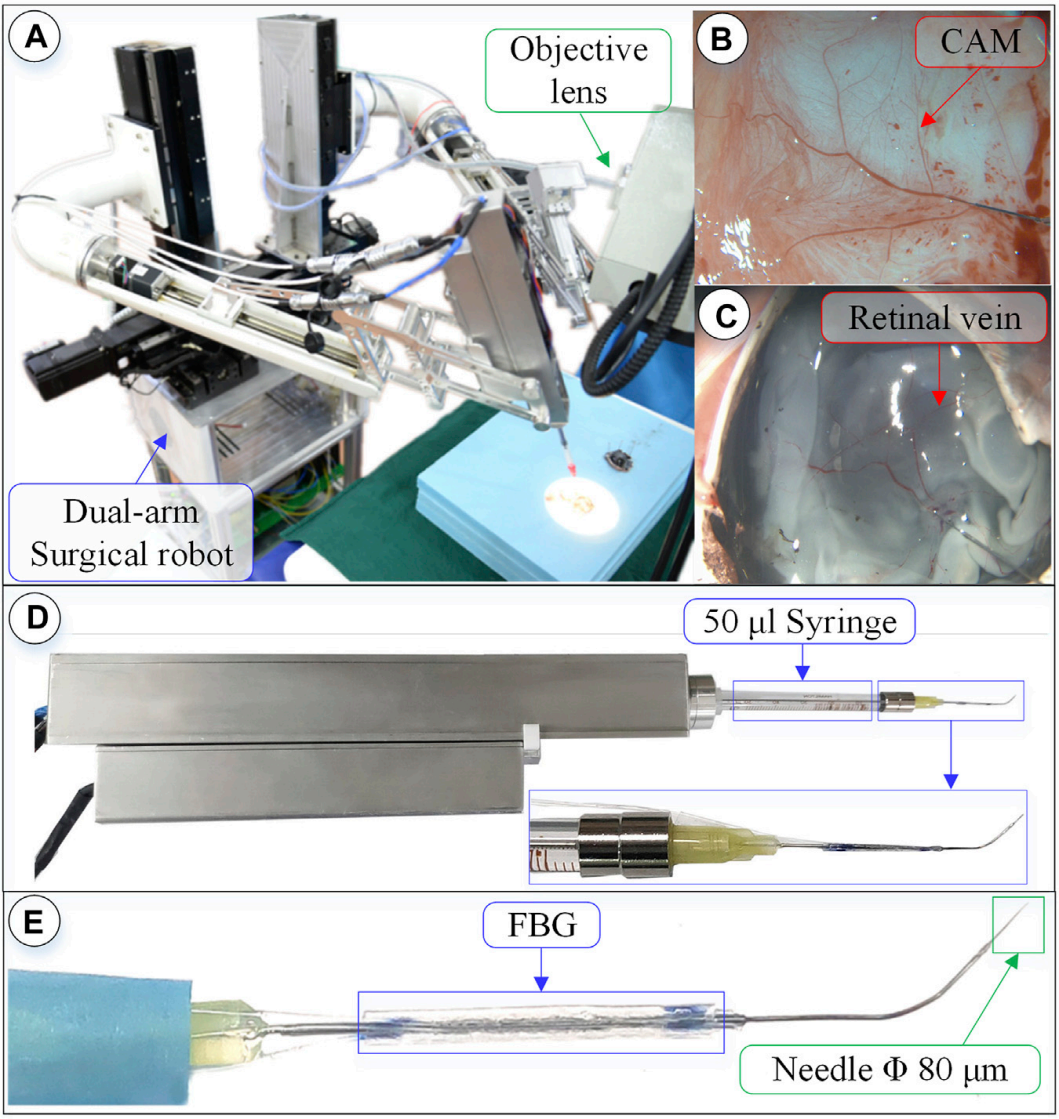
Surgical robot system under microscopic image. **(A)** Dual-arm robot under microscope. **(B)** CAM blood vessel. **(C)** Isolated pig eyeball retinal vein. **(D)** 3-DOF micro-force-sensing injection device. **(E)** Three-dimensional micro-force-sensing injection sleeve.

The robot-assisted retinal vein vascular injection platform consists of a self-developed dual-arm robot and the above injection instrument, an ophthalmic surgical microscope (Shanghai EDER Medical Equipment, SM-2000L), a 4K-3CMOS camera (SCIMEN, MDC-S150, Japan), a 3D display (SONY Active Shutter 3D, KD-65Z9D, Japan), main hand interactive device (Force-Dimension, Omega 6 ) and control host (CPU: Intel Core i9 10850K, Motherboard: Gigabyte Z490-UD, Memory: CORSAIR DDR4 16G 3000 high-frequency memory, hard disk: Samsung PM981 512G M.2 NVME high-speed solid state, graphics card: ASUS RTX2080TI-11G), and other components, as shown in Figure 6. The parameters of the multi-DOF dual-arm robot are listed in Table 1. The injection sleeve is fixed on the end of the right arm of the dual-arm robot through the quick installation interface. The operator wears shutter-type 3D glasses and controls the *x*, *y*, *z* linear motion of the robot in the three-dimensional space through the (6 + 1)-DOF main hand under the stereoscopic presentation of the microscopic binocular images on the 3D display. Reach the eyeball entry point, and then adjust the pitch (Eastern Motor, PKP223D15A-SG7.2), roll (Eastern Motor, PK543AW-A46), and feed (NiMotion, STM2851A-canopen) DOF of the robotic arm through the remote center of motion mechanism to determine the entry point. Through the demodulator, real-time collection and calculation of the wavelength change of the reflected light from the FBG sensor are used to identify the jump force signal when the tip of the instrument pierces through the blood vessel. After the tip of the instrument enters the blood vessel, the drug is advanced and released through the clamping freedom of the main hand. Among them, the maximum delivery speed of the injection instrument is 20 mm/s, the minimum speed is 5 μm/s, the roll angle accuracy is 0.5°, the drug advancing speed is up to 10 mm/s. After the push rod is in contact with the injection piston rod, the pushing accuracy is 0.01 mm, as shown in Figure 7.

**TABLE 1:** Parameters of the multi-DOF dual-arm robot.

Parameter	Component	Value
Motion range	*X*-axis	−30 ∼ + 30 mm
	*Y*-axis	−27 ∼ + 27 mm
	*Z*-axis	−60 ∼ + 60 mm
	Shoulder roll	±180°
	RCM pitch	±60°
Accuracy	X/Y/Z drive	10 *μ*m
Motion speed	Approach	≤10mm/s
	Insertion	≥0.5mm/s
	RCM adjustment	≤20°/s

### 4.1 Microscopic image-guided experiments of puncture and injection for retinal vein in isolated porcine eyeball

Pig eyes have a high degree of tissue similarity with human eyes and are easier to obtain, making them preferred experimental objects. There are 2–4 clearly visible veins in the fundus of fresh pig eyeballs; the diameter of which are between 80 and 200 μm. By taking 10 isolated pig eyeballs that have been slaughtered within 24 h, an incision is made from the middle of the sclera, the anterior segment of the eye is removed, and the vitreous body is taken out with forceps. Fix the fundus on the test board with three pins, and perform the retinal vascular puncture injection experiment.

Experiments on pig eyeballs are conducted as follows. (1) Determine the diameter of the target blood vessel, which is compared to a reference needle with an outer diameter of 200 μm. (2) Blood vessel alignment: operate the dual-arm robot to position the end of the injection device above the target blood vessel, and keep the angle between 15° and 30° with the blood vessel, which is convenient for blood vessel puncture. (3) Linear feed: control the linear feed of the injection mechanism so that the sleeve needle contacts the blood vessel. (4) Adjust the angle to reduce the angle between the needle tip and the blood vessel so that the needle tip is closer to the blood vessel wall. The needle tip is aligned with the central axis of the blood vessel, and the linear feed is continued until the puncture. (5) Stop feeding after piercing through the blood vessel and manipulate the automatic injection mechanism to inject the t-PA solution. (6) After the injection is completed, the drug injection sleeve is withdrawn.

**FIGURE 8:**
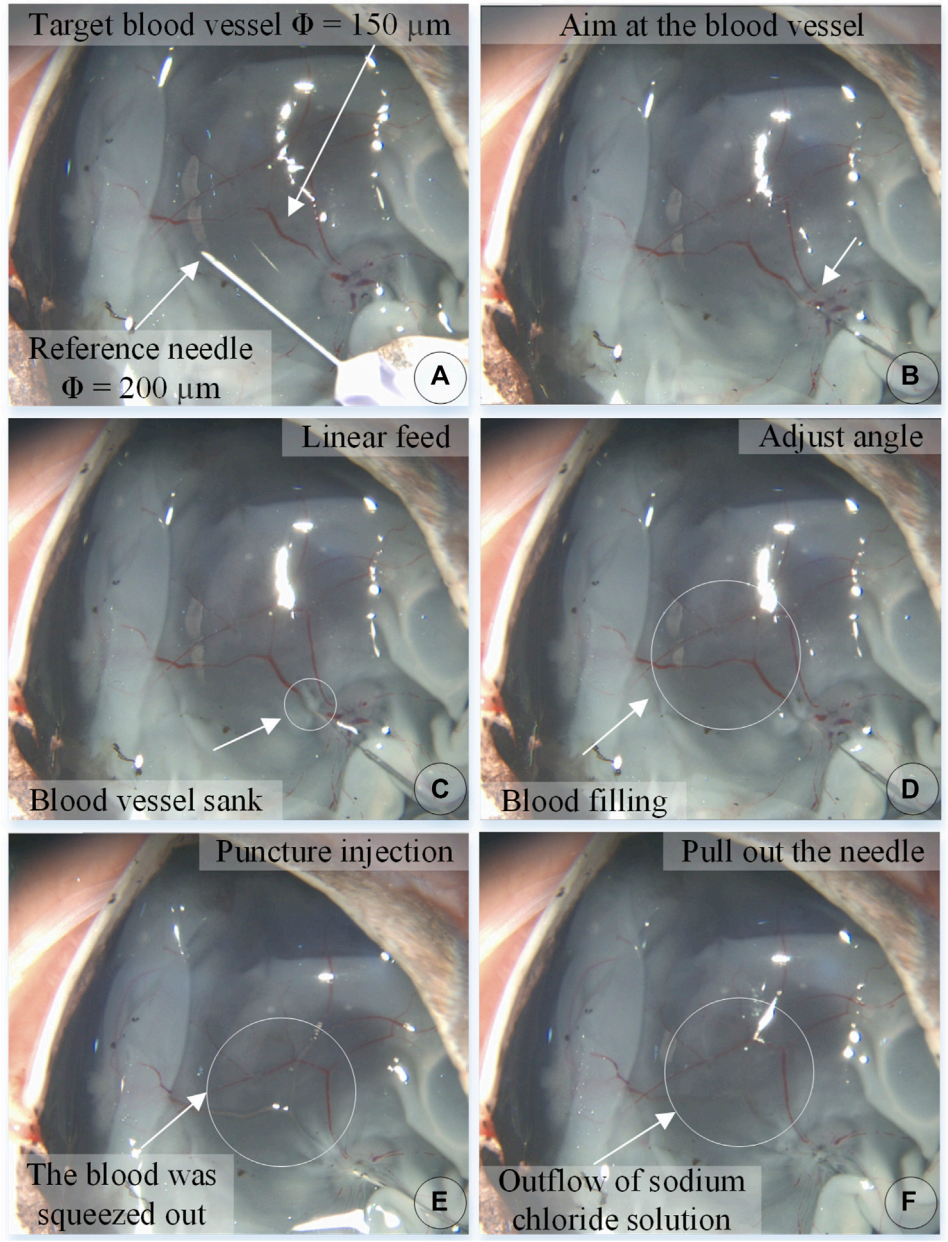
The puncture, injection and withdraw process of the isolated porcine eyeball retinal vein by microscopic images **(A–F)**.

Figure 8 shows an experiment of puncture injection into the retinal vein of the isolated porcine eyeball. (a) Determine that the target blood vessel of this puncture is about 150 μm. (b) Align the blood vessel and adjust the position of the sleeve needle and the blood vessel. (c) By feeding in a straight line, it can be observed that the needle presses down the blood vessel, the blood vessel sinks, the needle moves down for a certain distance, the blood at the puncture point of the blood vessel is squeezed away, and the blood vessel appears white. (d) Adjust the angle so that the needle tip goes deep into the blood vessel to facilitate puncture, and at this time, the blood in the target blood vessel is filled. (e) Puncture and injection: it can be observed that the blood vessel is washed away and appears transparent. (f) After the injection is completed, the sleeve needle is withdrawn, and it can be observed that some medicine liquid flows out from the puncture port.

**FIGURE 9:**
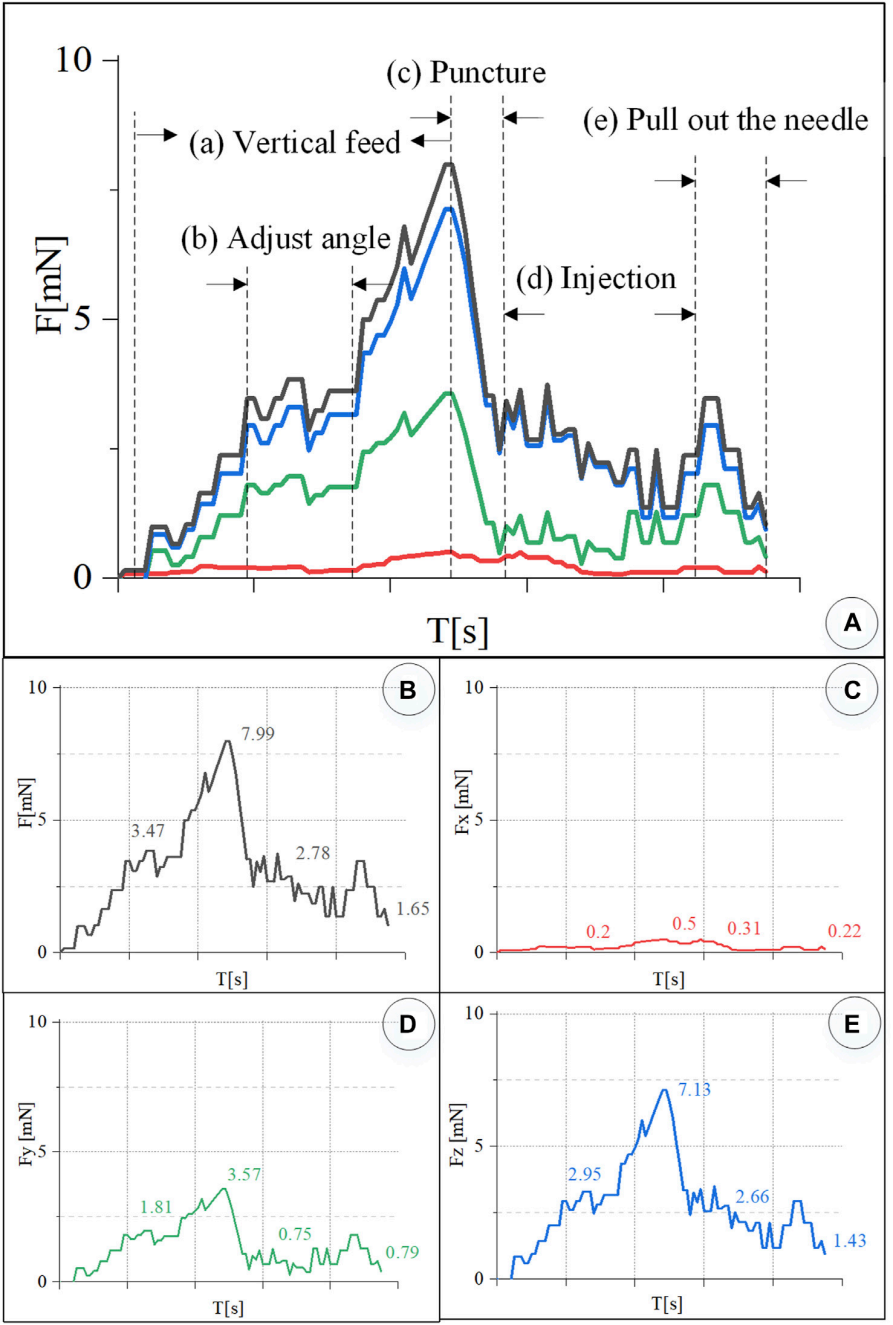
Real-time three-dimensional micro-force curve of retinal vein puncture injection of the isolated porcine eyeball. **(A)** Puncture process is divided into linear feed, angle adjustment, puncture, injection, and sleeve needle withdrawal stages. **(B–E)** Real-time curve of force *F*, *F*_*x*_, *F*_*y*_, *F*_*z*_, respectively.

In the experiment, the information on the wavelength change of the reflected light by the FBG sensor was recorded simultaneously and converted into real-time force data through the *K* matrix. Figure 9A shows the contact force curve of this experiment. After the injection sleeve is aligned with the blood vessel, the demodulator is reset to zero. The injection process is divided into five stages. (a) In the linear feed stage, the change in the wavelength of the reflected light by the FBG sensor gradually increases as the needle contacts the blood vessel, and the contact force continues to rise. (b) During the angle adjustment stage, the wavelength of the reflected light by the FBG sensor has a small change, and the contact force maintained at 3.74 mN. (c) During the puncture stage, the wavelength of the reflected light by the FBG drops sharply in a short time, and the contact force drops sharply. It can be judged that a puncture has occurred, and the feeding motion is stopped at this time. The puncture force is 7.99 mN. (d) In the injection phase, after the puncture occurs, the robot and the drug injection mechanism are kept at this position to start the drug delivery, and the contact force slowly drops to 2.78 mN. (e) At the stage of withdrawing the sleeve needle, the contact force rises for a period of time and then gradually drops to zero.

Figures 9B–D is the curve of the resultant force *F*, and *F*_*x*_, *F*_*y*_, and *F*_*z*_. The component force of the resultant force *F* in the *X* direction is very small, and the puncture force at the maximum moment is only 0.5 mN. The resultant force F is the largest in the *Z* direction. During the angle adjustment phase, the force F fluctuates up and down around 2.95 mN. The peak force in the puncture phase is 7.13 mN. The holding force during the injection phase is 2.66 mN. The force dropped to 1.43 mN during the withdrawal phase. *F* has a larger force component in the *Y* direction, and the forces in the four stages are 1.81, 3.57, 0.75, and 0.79 mN, respectively.

### 4.2 Microscopic image-guided CAM vascular puncture and injection experiments

The chicken embryo hatched 11–12 days of incubation from the fertilized egg contains a rich blood vessel membrane, called a chicken chorioallantoic membrane, hereinafter referred to as CAM, which appears like the retina’s vascular network structure. Its blood vessel diameter can be up to 500 μm, which can be used as the object of the puncture and injection experiment. The embryonic tissue at the bottom of the blood vessel is very soft, making it insufficient to support the blood vessel, and the puncture process is very difficult. Therefore, during dissection, the tissue in the eggshell is peeled off so that the complete CAM blood vessel remains on the eggshell. Under the premise of not affecting the vascular characteristics of CAM, the eggshell will support the blood vessel, which is convenient for the implementation of puncture injection.

Take 12-day fertilized chicken embryos, use a needle to pierce the top corner of the air chamber at the blunt end of the egg, and use tweezers and scissors to remove the outer shell of the air chamber without damaging the eggshell membrane to form an egg window. Because there are a few blood vessels on the eggshell membrane, the diameter of the blood vessels varies from 40 to 500 μm.

In order to tear off the eggshell membrane without damaging the blood vessels on the eggshell membrane, a small amount of sodium chloride solution is injected into the eggshell membrane to make the eggshell membrane transparent and the blood vessels on the eggshell membrane obvious. Look for a place where there is no blood vessel to insert the injection needle. Inject the sodium chloride solution into the eggshell membrane to bulge and separate it from the blood vessels. Use forceps to tear off the eggshell membrane. Evacuate the inner tissue of the chicken embryo, leaving the eggshell, modify the size of the eggshell according to the remaining CAM of the eggshell, and fix it under the microscope to wait for the puncture injection.

**FIGURE 10:**
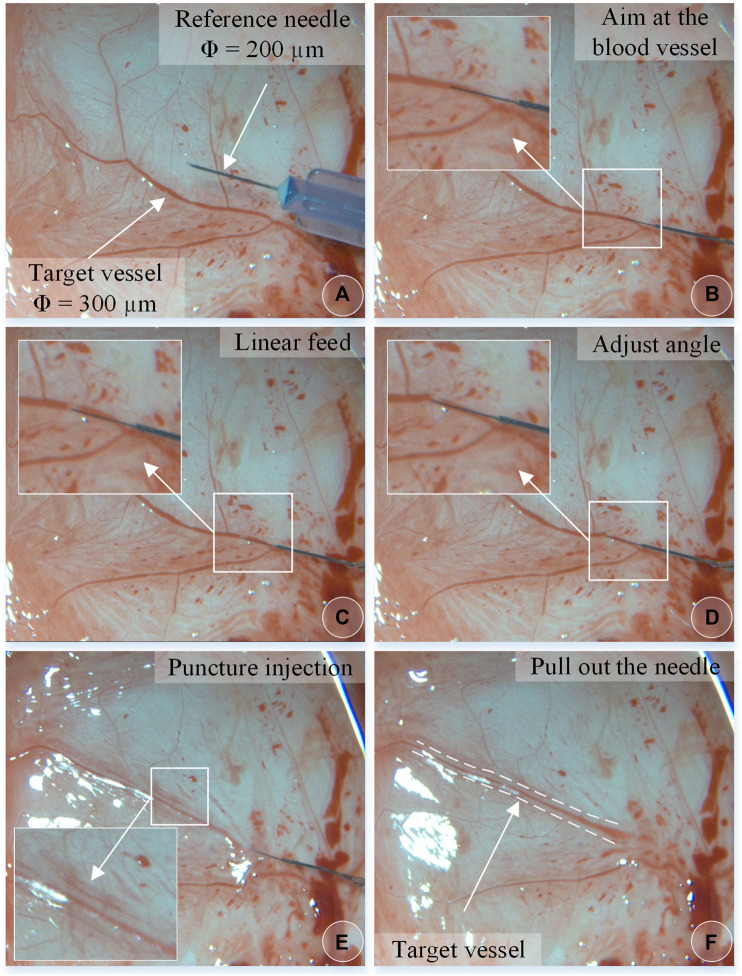
The vascular puncture, injection, and withdraw process of CAM by microscopic images **(A–F)**.

The experiment steps are the same as above. Figure 10 shows the chicken embryo puncture and injection experiment process. (a) Determine the target blood vessel for this puncture, with a diameter of about 300 μm. (b) Align the blood vessel, and adjust the position of the sleeve needle to the blood vessel. (c) By feeding straight, the needle presses down the blood vessel, the blood vessel sinks, the needle moves down for a certain distance, the blood at the puncture point of the blood vessel is squeezed away, and the blood vessel appears white. (d) Adjust the angle and pierce the needle into the blood vessel. (e) The sleeve structure remains stable, the medicine is automatically pushed and injected, and it can be observed that the blood vessel is washed away. (f) After the injection is completed, withdraw the needle. It can be observed that part of the t-PA solution flows out of the puncture port, and some blood backflows.

**FIGURE 11:**
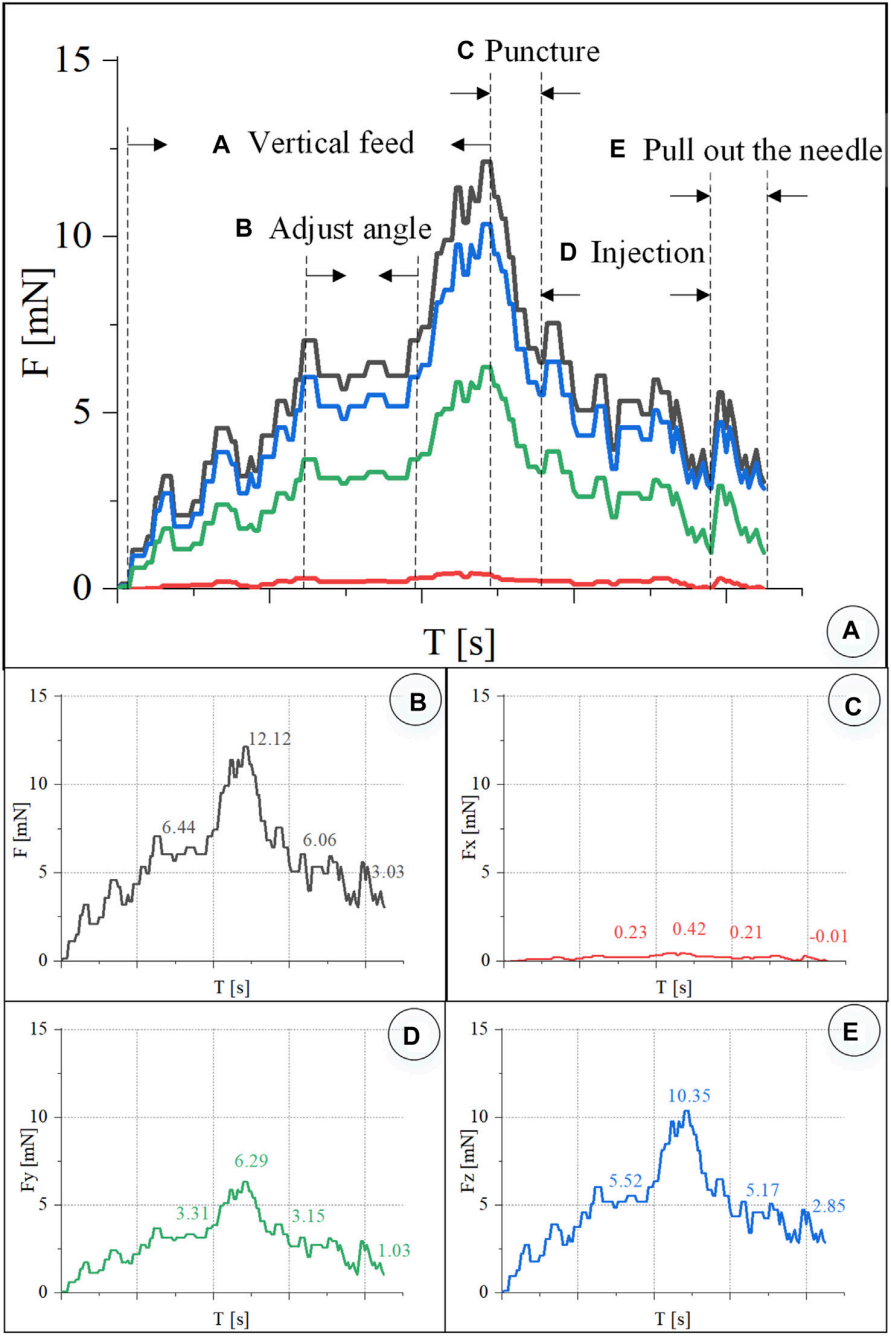
Real-time three-dimensional micro-force curve of CAM vascular puncture and injection. **(A)** Puncture process is divided into linear feed, angle adjustment, puncture, injection, and withdrawal of the sleeve needle. **(B–E)** Real-time curve of *F*, *F*_*x*_, *F*_*y*_, *F*_*z*_, respectively.

In the experiment, real-time recording of FBG reflected light wavelength change information was converted into real-time force data, as shown in Figure 11. The trend of the contact force between the injection sleeve and the blood vessel of the chicken embryo is the same as the change of the contact force between the retinal blood vessel from the porcine eyeball. The forces at each stage are as follows: the posture adjustment stage was 6.44 mN, the puncture stage was 12.12 mN, the injection stage was 6.06 mN, and the sleeve needle was withdrawn at 3.03 mN. The forces in the *X*, *Y*, and *Z* directions of the puncture stage are 0.42, 6.29, and 10.35 mN, respectively.

### 4.3 Repeat experiments

**FIGURE 12:**
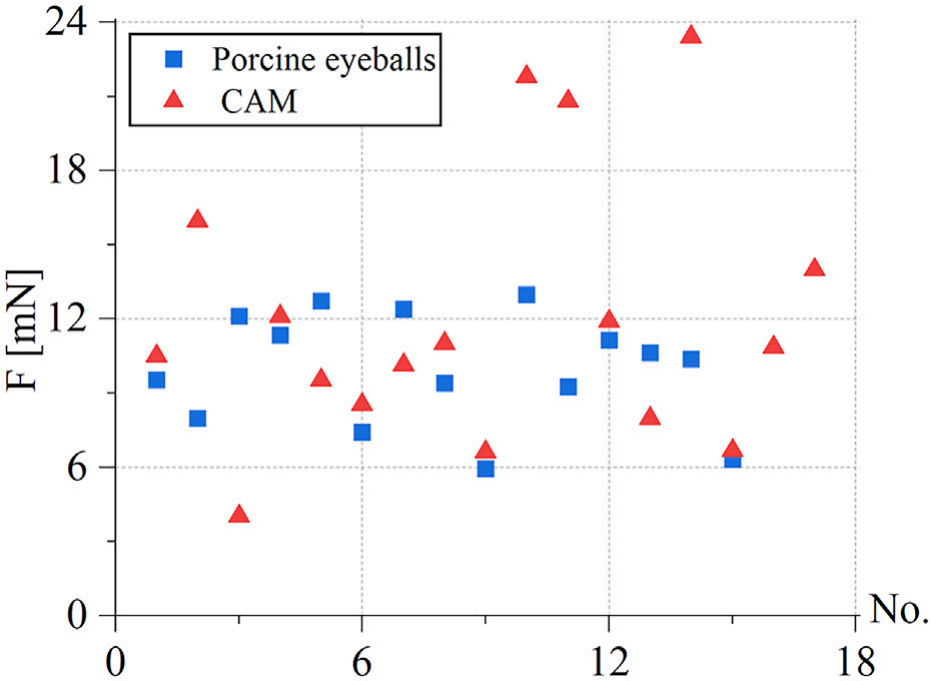
Statistics of puncture force for the retinal vein of 15 groups of pig eyes: 5.95**–**12.97 mN, 17 groups of CAM vascular: 4.02**–**23.4 mN.

Under the same environmental conditions, the robot was controlled to inject 10 isolated pig eyeballs. For each eyeball, two veins with a diameter of 100–200 μm were selected for injection, and 15 groups of three-dimensional contact force data at various stages were obtained. The puncture force range was 5.95–12.97 mN, and the average puncture force of the 15 groups was 9.98 mN. In the same way, 20 chicken embryos were injected, each blood vessel with a diameter of 150–500 μm was injected once, and 17 sets of puncture data were obtained. The puncture force range was 4.02–23.4 mN, and the average puncture force of the 17 groups was 12.05 mN, as shown in Figure 12.

During the experiment, blood vessels with large diameters and blood vessels that go straight were easier to puncture. The posture adjustment of the sleeve needle during the puncture process was the critical factor to the success of the puncture, and the proper posture was conducive to the insertion of the needle into the blood vessel. The real-time detection of the contact force of the reflected light wavelength change information conversion could effectively avoid the double puncture phenomenon. Therefore, robot-assisted fundus vascular injection has the advantage of smaller amplitude adjustment and higher sensitivity perception than manual operation. Due to the small blood vessels and the small volume of injected drugs, bleeding occurred in individual experiments.

### 4.4 Results analysis and discussion

Aiming at the clinical scenarios in which the retinal blood vessel diameter is usually less than 200 μm, this study proposed a cascade structure drug injection device for robot-assisted RVC surgery, which could achieve puncture and injection of blood vessels with a minimum of 100 μm. Patel et al. (2020) used a puncture needle tube with an outer diameter of 110 μm, and 200–410 μm CAM blood vessels were selected for puncture and injection in the experiment. In particular, this article designed an automatic drug injection mechanism, which could realize long-term and stable automatic drug delivery. In addition, the separate design consideration of micro-injector, injection sleeve, and driving mechanism was proposed in this article, making the drive mechanism completely encapsulated. The microinjection could be substituted according to the type and concentration of the drug prescribed. Replace the disposable consumables in time, and the injection sleeve can be sterilized separately at low temperature, which is convenient for bacteria isolation and is more in line with clinical operating specifications.

In terms of force-sensing methods, this study proposed a three-dimensional micro-force-sensing method with a ring array of FBG sensors embedded in the hollow outer wall of a nickel-titanium alloy tube. The interaction force between the tip of the sleeve and the soft tissue of the blood vessel could be restored more objectively. The RMS errors of the measured and actual forces in the three directions were 0.24, 0.18, and 1.71 mN, respectively. The RMS errors of the measured and actual values of the two-dimensional and three-dimensional force are 0.3 and 1.73 mN, respectively. Due to the small axial deformation of the injection sleeve, it is difficult to detect the axial force. At present, only two-dimensional force-sensing results are compared. The RMS error between the measured and actual values obtained in the literature (He et al., 2020a) is 0.70 mN.

Regarding the criteria for a successful vascular puncture, due to the limited resolution at the microscopic scale of optical imaging, coupled with the loss of image quality caused by illumination, transmission, display, and so on, the faint changes of the punctured blood vessel in the small pixel range on the image are difficult to identify. Therefore, real-time measurement of puncture force is particularly important. Because the deformation caused by force in the *Z* direction is very small, it is difficult to detect the real-time deformation in the *Z* direction through the FBG sensor. The literature (Gonenc and Iordachita, 2016; Gonenc et al., 2018; Patel et al., 2020; He C. et al., 2019; Vander Poorten et al., 2020; He et al., 2020a,b) mainly regarded the combined force of the needle tube in the *X* and *Y* directions as the puncture force. He X. et al. (2019) created a curved section to reduce the stiffness of the distal part of the instrument shaft to increase the deformation in the *Z* direction and placed the FBG sensor in the center of the needle tube to directly measure the force in the *Z* direction. For channel-type devices that require liquid injection, these two methods are not applicable. In this study, the injection sleeve was designed as a double-rigidity hollow structure, which increased the deformation in the *Z* direction. The relationship between the force in the *X* and *Y* directions and the wavelength change of the reflected light by the FBG sensor was obtained by the direct calibration method. Then, through the indirect calibration method, the relationship between the force in the *Z* direction and the wavelength change of the reflected light of the FBG sensor was obtained. The resultant force F was obtained through the coefficient matrix obtained by calibration. The experimental results showed that the direction of the force was mainly focused in the Y-Z plane, and the changing trend of the force in the three directions was the same. The force in the *Z* direction was the largest, followed by the *Y* direction, and the force in the *X* direction was close to 0 mN. Therefore, the resultant force F at this time could more objectively and directly restore the interaction force of blood vessels.

In terms of experimental methods, Yu et al. (2015) used rubber and agar to conduct experiments with self-made eyeballs, which was difficult to accurately simulate the real environment of the eye. Gonenc et al. (2018) and Patel et al. (2020) selected suspended CAM blood vessels for experiments. Although the liquid could also be successfully injected into the blood vessels, the suspended CAM blood vessels had no support and insufficient stability and were affected by the physiological pulsation of chicken embryos, which added interference factors and increased the difficulty of the experiment. This article used the retinal veins of the isolated porcine eyeball and the blood vessels on the eggshell to carry out the experiment. The retina of the isolated pig eyeball is more similar to the retina tissue of the human eye, which provided a suitable experimental model. The blood vessels on the eggshell are more obvious, and the eggshell will support the blood vessels, making the injection process more stable. This study lays the foundation for the experimental study of thrombolysis by injection of t-PA drugs under different conditions in the future.

Ourak et al. (2019) measured the puncture force in the isolated pig eyeball from 0.6 to 17.5 mN. A total of 80% of the puncture force was less than 7 mN. The reason was that too many punctures had been performed on an isolated pig eyeball, which caused excessive damage to the blood vessel tissue. In this work, only 1–2 puncture injections were performed on each isolated pig eyeball; thus, the measured puncture force range was 5.95–12.97 mN. Patel et al. (2020) measured an average puncture force of 13 mN in the CAM blood vessel. In this work, the average puncture force of the blood vessels of the 17 groups of CAM measured was 12.05 mN.

This article also has limitations. First, it is necessary to explore the influence of drug injection parameters on the thrombolysis effect, including physical parameters such as injection position, speed, and angle. The second is to explore more dexterous micromanipulation mechanisms that can reach more vascular locations in restricted entry points and intraocular manipulation spaces.

## 5 Conclusion

Based on the clinical requirements of RVC surgery, this research proposed a microscope image guidance robot-assisted high-precision retinal vein drug injection device. The designed dual-rigidity cascade drug injection sleeve with decoupled drive mechanism and automatic injection of thrombolytic drug mechanism could achieve 100–200 μm blood vessel puncture and stable drug injection, which improved the accuracy of the blood vessel puncture and drug injection operations under microscope magnified image. A three-dimensional force-sensing structure with a circular array of FBG sensors embedded in a hollow nickel-titanium alloy tube was proposed, which realized the real-time sensing of puncture force during retinal vein injection. Finally, the injection experiment on CAM and retinal vein of the isolated porcine eyeball were carried out with microscopic image guidance and a robot. The average puncture forces of the retinal vein vessels of the isolated porcine eyeball and CAM were 9.98 and 12.05 mN, respectively. This work verified the feasibility and effectiveness of microscopic image guidance robot-assisted RVC surgery *in vitro*, providing a reference for treating retinal vascular embolization under the assistance of robots.

## Data Availability

The raw data supporting the conclusion of this article will be made available by the authors without undue reservation.
